# Impact of the caFFR-Guided Functional SYNTAX Score on Ventricular Tachycardia/Fibrillation Development in Patients With Acute Myocardial Infarction

**DOI:** 10.3389/fcvm.2022.807805

**Published:** 2022-04-12

**Authors:** Jiazhi Pan, Qiuxia Zhang, Li Lei, Yaode Chen, Guodong Li, Hongbin Liang, Junyan Lu, Xinlu Zhang, Yongzhen Tang, Jun Pu, Yining Yang, Dapeng Mo, Jiancheng Xiu

**Affiliations:** ^1^Department of Cardiology, Nanfang Hospital, Southern Medical University, Guangzhou, China; ^2^Department of Cardiology, Zengcheng Branch of Nanfang Hospital, Guangzhou, China; ^3^Department of Cardiology, Renji Hospital, Shanghai Jiaotong University School of Medicine, Shanghai, China; ^4^Department of Cardiology, The First Affiliated Hospital of Xinjiang Medical University, Ürümqi, China; ^5^Department of Tiantan Interventional Neuroradiology, Beijing Tiantan Hospital of Capital Medical University, Beijing, China

**Keywords:** acute myocardial infarction, ventricular tachycardia, ventricular fibrillation, functional SYNTAX score, caFFR

## Abstract

**Aims:**

To explore the relationship between the severity of coronary artery disease (CAD) and the occurrence of ventricular tachycardia/ventricular fibrillation (VT/VF) in patients with acute myocardial infarction (AMI).

**Methods:**

We retrospectively enrolled 705 patients with AMI, who were hospitalized and underwent percutaneous coronary intervention (PCI), in Nanfang Hospital from July 2017 to July 2020. Logistic regression analysis and backward stepwise approach were taken to select the correlation factors. The left and the receiver operating characteristic curves (ROC) analysis were plotted to observe the discriminative power of the SYNTAX score (SS)/caFFR-guided functional SS (FSS_caFFR_) on the incident VT/VF.

**Results:**

About 58 (8.2%) patients experienced life-threatening VT/VF. The FSS_caFFR_ (OR: 1.155; 95% CI: 1.047 to 1.273; *p* = 0.004) was an independent predictor of VT/VF after AMI. The ROC analysis showed that the discriminative power of FSS_caFFR_ on the incident VT/VF was significantly better than SS (0.759 vs.0.695, *p* < 0.0001). Patients with VT/VF were categorized into 2 groups according to the interval between the onset of AMI and the VT/VF. The logistic regression analysis revealed that FSS_caFFR_ was a significant independent correlation of early- and late-VT/VF.

**Conclusion:**

The incident VT/VF in patients with AMI is closely associated with the severity of CAD evaluated by SS and FSS_caFFR_. Compared to SS, FSS_caFFR_ has a higher correlation with VT/VF, and FSS_caFFR_ was demonstrated to be an independent correlation factor of incident VT/VF after AMI.

## Introduction

Ventricular tachycardia/ventricular fibrillation (VT/VF) is one of the most severe complications in patients with acute myocardial infarction (AMI). Patients with AMI who develop VT/VF often show long-term hospitalization and a poor prognosis (1). Early identification of individuals at high risk of VT/VF may improve the prognosis of patients with AMI. Although many recent studies have described the risk factors for VT/VF after AMI, the relationship between the severity of coronary artery disease (CAD) and the occurrence of VT/VF in patients with AMI remains unclear.

The SYNTAX score (SS) is an anatomical scoring system that indicates the severity of coronary artery lesions according to the number of lesions, their functional role, location, and complexity (2). However, in many cases, the anatomic lesion severity does not match the coronary physiology using fractional flow reserve (FFR). Therefore, SS, based solely on anatomical information, may lead to an erroneous assessment of lesion complexity, limiting the discriminant ability of the SS. Thus, in 2011, the concept of the functional SS (FSS) was developed (SS is only calculated in vessels with low FFR) by Nam et al., and they demonstrated that the FSS has a better prognostic implication compared to classic SS (3).

Computational pressure-flow dynamics-derived FFR (caFFR) is derived without using a pressure wire or hyperemia induction. A recent study has validated that caFFR has high accuracy, sensitivity, and specificity, with FFR as the reference standard (4). Considering that caFFR is more economical and safer than traditional FFR, FSS guided by caFFR (FSS_caFFR_) is a faster and more accurate indicator to assess the severity of the patient's coronary artery. To our best knowledge, no literature report has evaluated the relationship between FSS_caFFR_ and VT/VF.

Therefore, this study is aimed to investigate the impact of CAD severity on the incident VT/VF by using SS and FSS_caFFR_ to evaluate the coronary conditions in patients with AMI. It also aimed to explore which index could better guide the clinical practice to reduce the occurrence of VT/VF and improve the prognosis of patients with AMI.

## Methods

### Study Population

We retrospectively enrolled 759 patients with AMI, who were hospitalized and had undergone percutaneous coronary intervention (PCI) at Nanfang Hospital of Southern Medical University from July 2017 to July 2020. Patients who met any of the following conditions were excluded: received thrombolysis or stent implantation or coronary artery bypass grafting (CABG) treatment in the past; developed AMI that was not caused by coronary stenosis or occlusion (such as myocardial infarction with no obstructive coronary atherosclerosis (MINOCA), acute myocarditis, aortic dissection); diagnosed with atrial or ventricular arrhythmia before admission; had severe valvular heart disease or congenital heart disease; had a severe infection or advanced cancer, malignant tumors, and other diseases at the time of admission; or had missed or incomplete clinical data. The study protocol was reviewed and approved by the Local Ethics Committee of the Southern Medical University in accordance with the Declaration of Helsinki.

The acute myocardial infarction was defined as cardiomyocyte necrosis in a clinical setting, consistent with acute myocardial ischemia. A combination of criteria is required to meet the diagnosis of AMI—i.e., the detection of an increase and/or decrease in a cardiac biomarker, preferably high-sensitivity cardiac troponin (hs-cTn) T or I, with at least one value above the 99th percentile of the upper reference limit and at least one of the following: symptoms of myocardial ischemia; new ischemic ECG changes; development of pathological Q waves on ECG; imaging evidence of loss of viable myocardium or new regional wall motion abnormality in a pattern consistent with an ischemic etiology; intracoronary thrombus detected on angiography or autopsy (5). The VT/VF was defined as a fatal ventricular tachycardia or fibrillation that lasted longer than 30 s or required electrical cardioversion and/or antiarrhythmic drugs. The observation time was the period from the onset of AMI to hospitalization. Additionally, to study the related factors affecting the onset time of VT/VF, we divided the patients with VT/VF into two groups according to the interval between the onset of AMI and the VT/VF: within 0–48 h after the onset of AMI (the early-VT/VF group) and 48 h after the onset of AMI (the late-VT/VF group). The clinical features were compared with those of the patients with non-VT/VF for each group to clarify the independent correlation factors of VT/VF.

### Data Collection

In this study, the baseline characteristics, medical history data, and treatment strategy of patients were acquired through hospitalized medical records. Venous blood samples were collected from all the patients within 2 h after admission, and complete blood counts and blood biochemical parameters were measured. The peak N-terminal precursor brain natriuretic peptide (NT-proBNP), peak creatine kinase myocardial band (CK-MB), and peak cTnI of the patient were determined according to the blood biochemical results monitored during hospitalization. The estimated glomerular filtration rate (eGFR) was calculated by using the modification of diet in renal disease (MDRD) equation (6).

All the patients had completed echocardiographic testing within 48 h after admission and had undergone coronary angiography using the femoral or radial approaches. The procedure was performed by interventional cardiologists according to published guidelines, institutional policy, and routine practice.

Off-line caFFR assessment was performed by blinded experienced analysts certified in using the software with the FlashAngio system (including the FlashAngio console, FlashAngio software, and Flash Pressure transducer; Rainmed Ltd., Suzhou, China). The details of caFFR measurement were the same as those of the previously reported method (4). Fluoroscopic visualizations were analyzed by 2 independent and experienced interventional cardiologists who were blinded to all the data. In the case of disagreement on visual evaluation, the final decision was made by consensus. Each coronary (including the infarct-related artery) lesion that constituted luminal obstruction ≥ 1.5 mm in diameter and ≥ 50% stenosis was scored to count the SS using the online calculator version of 2.28 (www.syntaxscore.com). The FSS_caFFR_ was defined as modified SS measured only in lesions with a caFFR of ≤0.8.

### Statistical Analysis

The normal distribution of the data was tested using the one-sample Kolmogorov–Smirnov test. Continuous variables conforming to a normal distribution were expressed as means ± SD; otherwise, they were expressed as medians with an interquartile range. Categorical variables were summarized using proportions. Characteristics between the patients with and without VT/VF were analyzed using the chi-squared test for categorical variables and the *t*-test or the Mann–Whitney U test for continuous variables.

Variables that were statistically significant in the univariable logistic analysis were included in the multivariable logistic analysis. Stepwise backward likelihood ratio regression was performed to screen the variables by successively removing non-significant (*p* > 0.05) covariates until all the remaining variables were statistically significant. Notably, variables such as intra-aortic balloon pump (IABP), mechanical ventilation, temporary pacing, hemodialysis, and continuous renal replacement therapy (HD/CRRT) were not included in this analysis because they were more likely to be caused by VT/VF. Receiver operating characteristic curves (ROC) were plotted to show the power of SS and FSS_caFFR_ to predict incident VT/VF. In all analyses, *p* < 0.05 was considered statistically significant. All analyses were conducted using SPSS (V.22.0) and MedCalc (V.15).

## Results

### Population Characteristics

**Figure 1:**
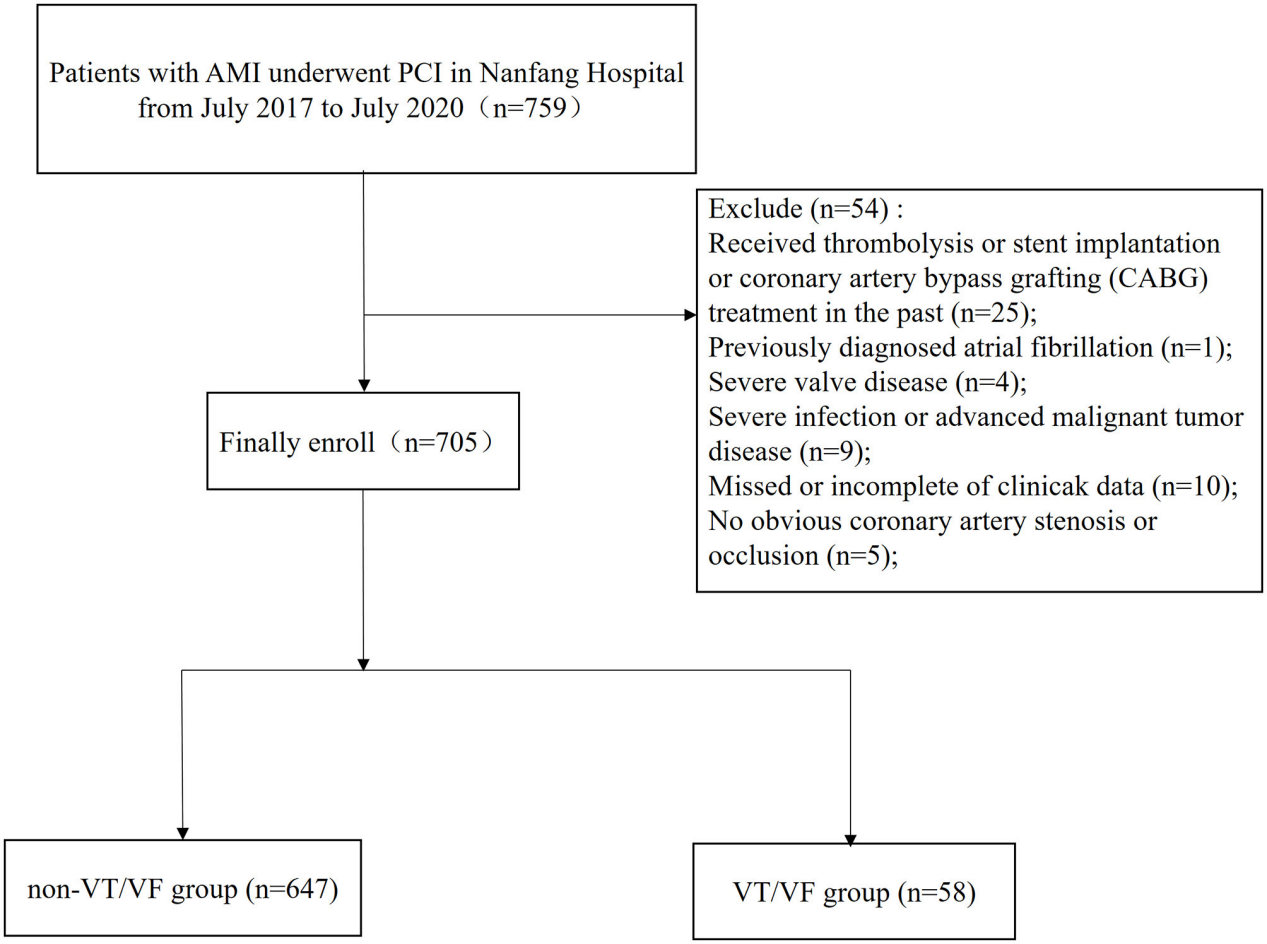
A flow chart of study design and participants.

Among the 705 included patients with AMI, 58 (8.2%) patients developed VT/VF (Figure 1). The details of these patients are listed in Table 1 and Supplementary Table 1.

**Table 1:** Baseline characteristics of patients in the non–VT/VT group and the VT/VF group.

**Variables**	**Non–VT/VF group (*n* = 647)**	**VT/VF group** **(*n* = 58)**	***p***
Age (years)	58 ± 13	62 ± 14	0.046
Male (%)	546 (82.8)	43 (71.4)	0.097
SBP (mmHg)	128 ± 20	114 ± 22	<0.001
DBP (mmHg)	80 ± 14	71 ± 16	<0.001
Heartrate (bpm)	80 ± 15	92 ± 23	<0.001
BMI (kg/m^2^)	24.4 ± 3.0	24.5 ± 2.7	0.790
Diabetes mellitus (%)	195 (30.1)	27 (46.6)	0.010
Hypertension (%)	368 (56.9)	28 (48.3)	0.206
Dyslipidemia (%)	295 (45.6)	29 (50.0)	0.519
COPD (%)	20 (3.1)	1 (1.7)	0.557
PAD (%)	61 (9.4)	8 (13.8)	0.284
Smoking (%)	380 (58.7)	33 (56.9)	0.786
STEMI (%)	391 (60.4)	49 (84.5)	<0.001
Killip class>I (%)	137 (21.2)	39 (67.2)	<0.001
Peak CK-MB (U/L)	27.695 (5.445–121.880)	84.180 (21.455–245.700)	<0.001
Peak CTnI (ng/ml)	19.378 (3.526–50.000)	50.000 (12.001–50.000)	0.002
Peak NT-ProBNP (pg/ml)	864 (230–2520)	3546 (976–8220)	<0.001
WBC (×10^9^/L)	10.59 ± 3.49	13.89 ± 5.83	<0.001
Hemoglobin (g/L)	141 ± 50	136 ± 22	0.517
LDL-c (mmol/L)	3.32 ± 1.06	3.48 ± 1.45	0.292
HDL-c (mmol/L)	1.05 ± 0.27	1.05 ± 0.29	0.967
Cholesterol (mmol/L)	4.97 ± 1.31	5.05 ± 1.69	0.692
CRP (mg/L)	4.185 (1.605–12.340)	6.835 (2.318–15.233)	0.050
Uric acid (μmol/L)	381 ± 112	452 ± 143	<0.001
HbAlc (%)	6.0 (5.7–6.8)	6.2 (5.8–7.1)	0.140
K^+^ (mmol/L)	4.01 ± 0.47	4.16 ± 0.56	0.018
Mg^2+^ (mmol/L)	0.84 ± 0.10	0.90 ± 0.20	<0.001
SCr (μmol/L)	100 ± 87	109 ± 36	0.437
eGFR (ml/min/1.73 m^2^)	83 ± 27	67 ± 24	<0.001
LVEF (%)	57.7 ± 7.8	48.3 ± 10.0	<0.001
LVEDD (mm)	45 ± 4	46 ± 6	0.124
LVA (%)	59 (9.1)	11 (19.0)	0.030
β-blocker (%)	446 (68.9)	30 (51.7)	0.007
CCB (%)	57 (8.8)	3 (5.2)	0.342
ACEIs/ARBs (%)	289 (44.7)	15 (25.9)	0.006
Diuretics (%)	123 (19.0)	32 (55.2)	<0.001
IABP (%)	28 (4.3)	24 (41.4)	<0.001
Mechanical ventilation (%)	7 (1.1)	24 (41.4)	<0.001
Temporary pacing (%)	11 (1.7)	10 (17.2)	<0.001
HD/CRRT (%)	9 (1.4)	5 (8.6)	<0.001
MI-PCI TIME interval (%)			0.314
<3 h	82 (12.7)	11 (19.0)	
3–12 h	246 (38.0)	26 (44.8)	
12–24 h	93 (14.4)	7 (12.1)	
24–72 h	88 (13.6)	7 (12.1)	
>72 h	138 (21.3)	7 (12.1)	
Infarct related artery (%)			
LAD	328 (50.7)	32 (55.2)	0.514
LCX	100 (15.5)	8 (13.8)	0.736
RCA	201 (31.1)	17 (29.3)	0.782
Other	22 (3.4)	2 (3.4)	0.985
LMCA disease (%)	59 (9.1)	15 (25.9)	<0.001
Post-TIMI grade <3 (%)	26 (4.0)	8 (13.8)	<0.001
Rentrop grade>1 (%)	87 (13.4)	12 (20.7)	0.186
Multivessel CAD (%)	479 (74.0)	45 (77.6)	0.678
Multivessel PCI (%)	79 (12.2)	7 (12.1)	0.904
Stents used per patient	1.09 ± 0.51	1.27 ± 0.64	0.039
SS	24.4 ± 11.7	32.6 ± 13.5	<0.001
FSS_caFFR_	18.0 ± 7.6	31.8 ± 9.1	<0.001

*SBP, systolic blood pressure; DBP, diastolic blood pressure; BMI, body mass index; COPD, chronic obstructive pulmonary disease; PAD, peripheral artery disease; STEMI, ST-segment elevation myocardial infarction; eGFR, estimated glomerular filtration rate; WBC, white blood cell; CK-MB, creatine kinase-myocardial band; cTnI, cardiac troponin I; NT-proBNP, N-terminal precursor brain natriuretic peptide; WBC, white blood cell; LDL-C, low-density lipoprotein cholesterol; HDL-C, high-density lipoprotein cholesterol; CRP, C-reactive protein; HbAlc, glycated hemoglobin; K^+^, potassium; Mg^2+^, magnesium; SCr, serum creatinine; eGFR, estimated glomerular filtration rate; LVEF, left ventricular ejection fraction; LVEDD, left ventricular end-diastolic dimension; LVA, left ventricular aneurysm; CCB, calcium channel blocker; ACEIs/ARBs, angiotensin-converting enzyme inhibitors/angiotensin receptor blockers; IABP, intra-aortic balloon pump; HD/CRRT, hemodialysis/continuous renal replacement therapy; LAD, left anterior descending; LCX, circumflex; RCA, right coronary artery; LMCA, left main coronary artery; TIMI, thrombolysis in myocardial infarction; CAD, coronary artery disease; PCI, percutaneous coronary intervention; SS, SYNTAX score; FSS_caFFR_, functional SYNTAX score guided by computational pressure-flow dynamics-derived fractional flow reserve*.

Compared with the non-VT/VF group, the patients with VT/VF had a higher Killip class and heart rate on admission and lower systolic blood pressure (SBP) and diastolic blood pressure (DBP). Patients with ST-elevation myocardial infarction (STEMI) and patients with diabetes were more likely to develop VT/VF. Regarding laboratory examinations, higher peak CK-MB, peak cTnI, peak NT-proBNP, white blood cell (WBC), C-reactive protein (CRP), uric acid, potassium (K^+^), magnesium (Mg^2+^), and lower eGFR values were found among patients who had developed VT/VF. Additionally, the patients with VT/VF had a lower LVEF and a higher incidence of the left ventricular aneurysm.

Regarding the therapies conducted in these patients, β-blockers and angiotensin-converting enzyme inhibitors/angiotensin receptor blockers (ACEIs/ARBs) were used less frequently among the patients with VT/VF. By contrast, the use of diuretics, IABP, mechanical ventilation, temporary pacing, and HD/CRRTwas higher in the patients with VT/VF.

Concerning coronary angiographic characteristics, the incidence of left main coronary artery (LMCA) disease and post-PCI TIMI Grade <3 was significantly higher in the VT/VF group. The patients who developed VT/VF had more coronary artery lesions and received more stents during the procedure. Notably, SS and FSS_caFFR_ were significantly higher in the patients with VT/VF than those without VT/VF [32.6 ± 13.5 vs. 24.4 ± 11.7 (*p* < 0.001) and 31.8 ± 9.1 vs. 18. ± 7.6 (*p* < 0.001), respectively].

### Correlation Factors of Incident VT/VF

The univariable logistic analysis date is detailed in Table 2. Using multivariable logistic analysis and the backward stepwise approach, SBP (OR: 0.969; 95% CI: 0.950 to.987; *p* = 0.001), STEMI (OR: 2.597; 95% CI: 1.048 to 6.452; *p* = 0.039), WBC (OR: 1.146; 95% CI: 1.064 to 1.234; *p* < 0.001), LVEF (OR: 0.952; 95% CI: 0.917 to.989; *p* = 0.011), diuretic (OR: 2.262; 95% CI: 1.074 to 4.785; *p* = 0.032), and FSS_caFFR_ (OR: 1.155; 95% CI: 1.047 to 1.273; *p* = 0.004) were independent correlation factors of incident VT/VF after AMI (Table 3).

**Table 2:** Univariable logistic regression analysis for relevant factors of VT/VF after acute myocardial infarction (AMI).

	**OR**	**95%Cl**	***p***
Age (years)	1.022	1.000–1.044	0.047
Male (%)	0.594	0.319–1.106	0.100
SBP (every increase 10 mmHg)	0.668	0.572–0.779	<0.001
DBP (every increase 10 mmHg)	0.640	0.518–0.791	<0.001
Heartrate (bpm)	1.040	1.025–1.055	<0.001
BMI (kg/m^2^)	1.012	0.925–1.108	0.789
Diabetes mellitus (%)	2.019	1.173–3.473	0.011
Hypertension (%)	0.708	0.413–1.212	0.208
Dyslipidemia (%)	1.193	0.697–2.042	0.519
COPD (%)	0.550	0.072–4.173	0.563
PAD (%)	1.537	0.697–3.392	0.287
Smoking (%)	0.927	0.539–1.596	0.786
Killipclass>I on admission (%)	7.641	4.279–13.646	<0.001
STEMI (%)	3.565	1.721–7.383	0.001
Peak CK**-**MB (U/L)	1.004	1.002–1.007	<0.001
Peak CTnI (ng/ml)	1.022	1.008–1.036	0.002
Peak NT**-**proBNP (every increase 100 pg/ml)	1.004	1.001–1.007	0.014
WBC (×10^9^/L)	1.200	1.128–1.276	<0.001
HGB (g/L)	0.994	0.981–1.007	0.360
LDL-c (mmol/L)	1.133	0.898–1.428	0.292
HDL-c (mmol/L)	0.980	0.365–2.632	0.967
Cholesterol (mmol/L)	1.040	0.856–1.263	0.692
CRP (mg/L)	1.005	0.999–1.011	0.127
Uric acid (μmol/L)	1.005	1.003–1.007	<0.001
HbAlc (%)	1.009	0.961–1.059	0.730
K^+^ (every increase 0.1 mmol/l)	1.067	1.012–1.126	0.017
Mg^2+^ (every increase 0.1 mmol/l)	1.393	1.157–1.678	<0.001
SCr (μmol/L)	1.001	0.998–1.003	0.444
eGFR (ml/min/1.73 m^2^)	0.978	0.968–0.988	<0.001
LVEDD (mm)	1.060	0.998–1.123	0.054
LVEF (%)	0.896	0.870–0.923	<0.001
LVA (%)	2.332	1.148–4.739	0.019
MI-PCI TIME	0.798	0.645–1.314	0.127
β-blocker (%)	0.483	0.281–0.830	0.008
CCB (%)	0.565	0.171–1.862	0.348
ACEIs/ARBs (%)	0.432	0.235–0.794	0.007
Diuretics (%)	5.243	3.015–9.120	<0.001
Infarct related artery (%)			
LAD	1.197	0.698–2.054	0.514
LCX	0.875	0.403–1.902	0.736
RCA	0.920	0.510–1.659	0.782
Other	1.015	0.233–4.427	0.985
LMCA disease (%)	3.477	1.823–6.632	<0.001
PostTIMI grade <3 (%)	3.822	1.645–8.880	0.002
Rentrop grade>1 (%)	1.679	0.856–3.296	0.132
Multivessel CAD (%)	0.869	0.448–1.686	0.678
Multivessel PCI (%)	0.950	0.417–2.167	0.904
Stents used per patient	1.653	1.027–2.660	0.039
SS	1.052	1.030–1.074	<0.001
FSS_caFFR_	1.073	1.051–1.086	<0.001

*SBP, systolic blood pressure; DBP, diastolic blood pressure; BMI, body mass index; COPD, chronic obstructive pulmonary disease; PAD, peripheral artery disease; STEMI, ST-segment elevation myocardial infarction; eGFR, estimated glomerular filtration rate; WBC, white blood cell; CK-MB, creatine kinase-myocardial band; cTnI, cardiac troponin I; NT-proBNP, N-terminal precursor brain natriuretic peptide; WBC, white blood cell; LDL-C, low-density lipoprotein cholesterol; HDL-C, high-density lipoprotein cholesterol; CRP, C-reactive protein; HbAlc, glycated hemoglobin; K^+^, potassium; Mg^2+^, magnesium; SCr, serum creatinine; eGFR, estimated glomerular filtration rate; LVEF, left ventricular ejection fraction; LVEDD, left ventricular end-diastolic dimension; LVA, left ventricular aneurysm; CCB, calcium channel blocker; ACEIs/ARBs, angiotensin-converting enzyme inhibitors/angiotensin receptor blockers; IABP, intra-aortic balloon pump; HD/CRRT, hemodialysis/continuous renal replacement therapy; LAD, left anterior descending; LCX, circumflex; RCA, right coronary artery; LMCA, left main coronary artery; TIMI, thrombolysis in myocardial infarction; CAD, coronary artery disease; PCI, percutaneous coronary intervention; SS, SYNTAX score; FSS_caFFR_, functional SYNTAX score guided by computational pressure-flow dynamics-derived fractional flow reserve*.

**Table 3:** Multivariable logistic regression analysis for independent predictors of VT/VF after AMI.

	**OR**	**95%Cl**	***p***
SBP (every increase 10 mmHg)	0.969	0.950–0.987	0.001
STEMI (%)	2.597	1.048–6.452	0.039
WBC (×10^9^/L)	1.146	1.064–1.234	<0.001
LVEF (%)	0.952	0.917–0.989	0.011
Diuretics (%)	2.262	1.074–4.785	0.032
FSS_caFFR_	1.155	1.047–1.273	0.004

*SBP, systolic blood pressure; STEMI, ST-segment elevation myocardial infarction; WBC, white blood cell; LVEF, left ventricular ejection fraction; FSS_caFFR_, functional SYNTAX score guided by computational pressure-flow dynamics-derived fractional flow reserve*.

### The Discriminative Power of SS/FSS_caFFR_ and Risk Stratification of VT/VF

To further explore the relationship between SS/FSS_caFFR_ and VT/VF, 705 patients were divided into tertiles (intertertile range: 19 to 28.5) of risk based on SS, i.e., low, medium, and high SS (33.6%, *n* = 237; 32.8%, *n* = 231; 33.6%, *n* = 237, respectively), and were analyzed. After calculating FSS_caFFR_, 22.4% of the high-risk SS group moved to the medium-risk FSS_caFFR_ group and 13.1% moved to the low-risk FSS_caFFR_ group, whereas 37.6% of the medium-risk SS group moved to the low-risk FSS_caFFR_ group (Supplementary Figure 1).

The VT/VF occurred in 3.4, 6.9, and 14.3% of patients with low, medium, and high SS, respectively (*p* < 0.0001). A similar trend was observed in the FSS_caFFR_ group (2.3, 10.2, and 19.6% in the low-, medium-, and high-FSS_caFFR_ groups, respectively) (*p* < 0.0001) (Supplementary Figure 2).

**Figure 2:**
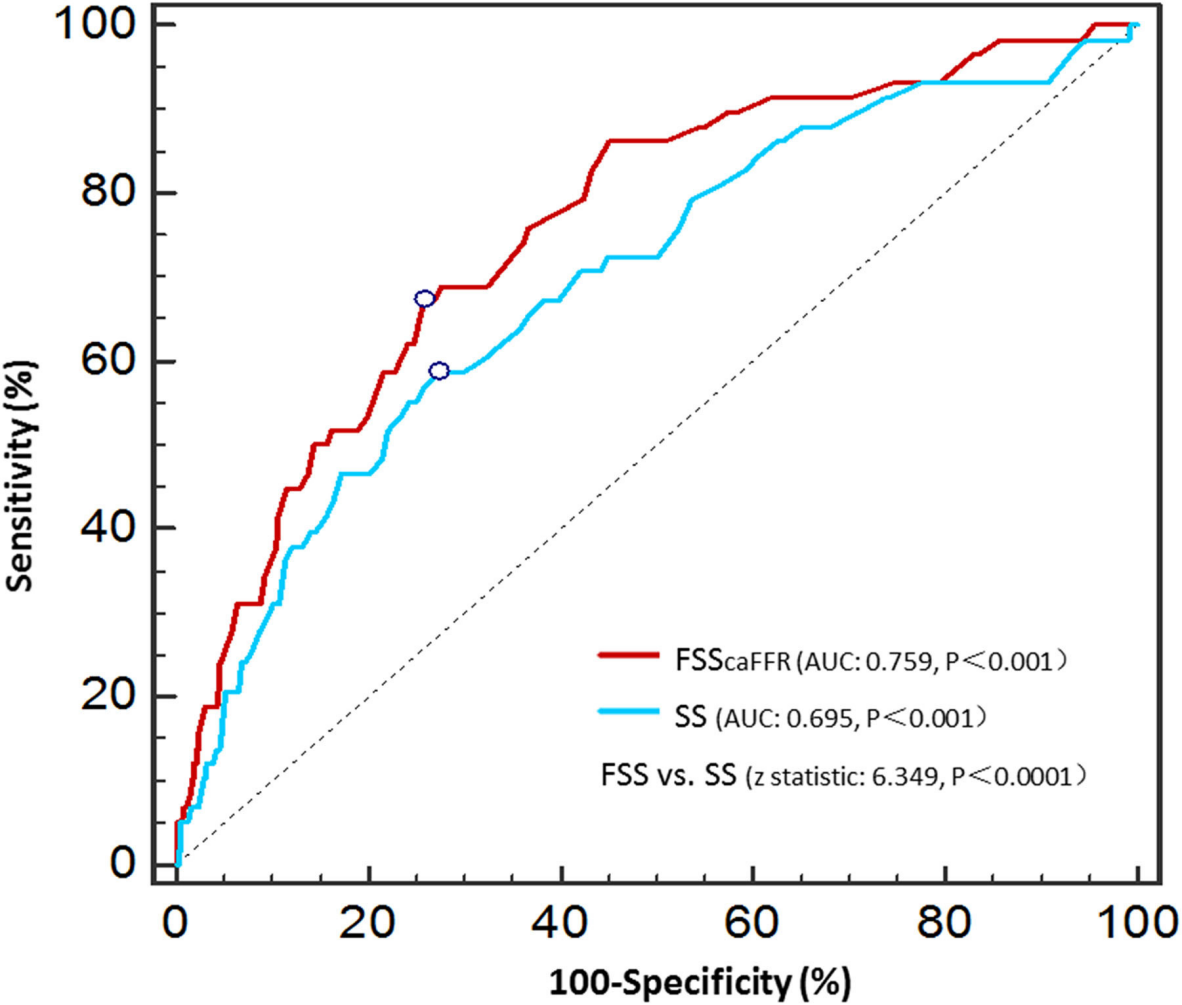
Receiver operator characteristic curves of the SS and FSS_caFFR_.

**Figure 3:**
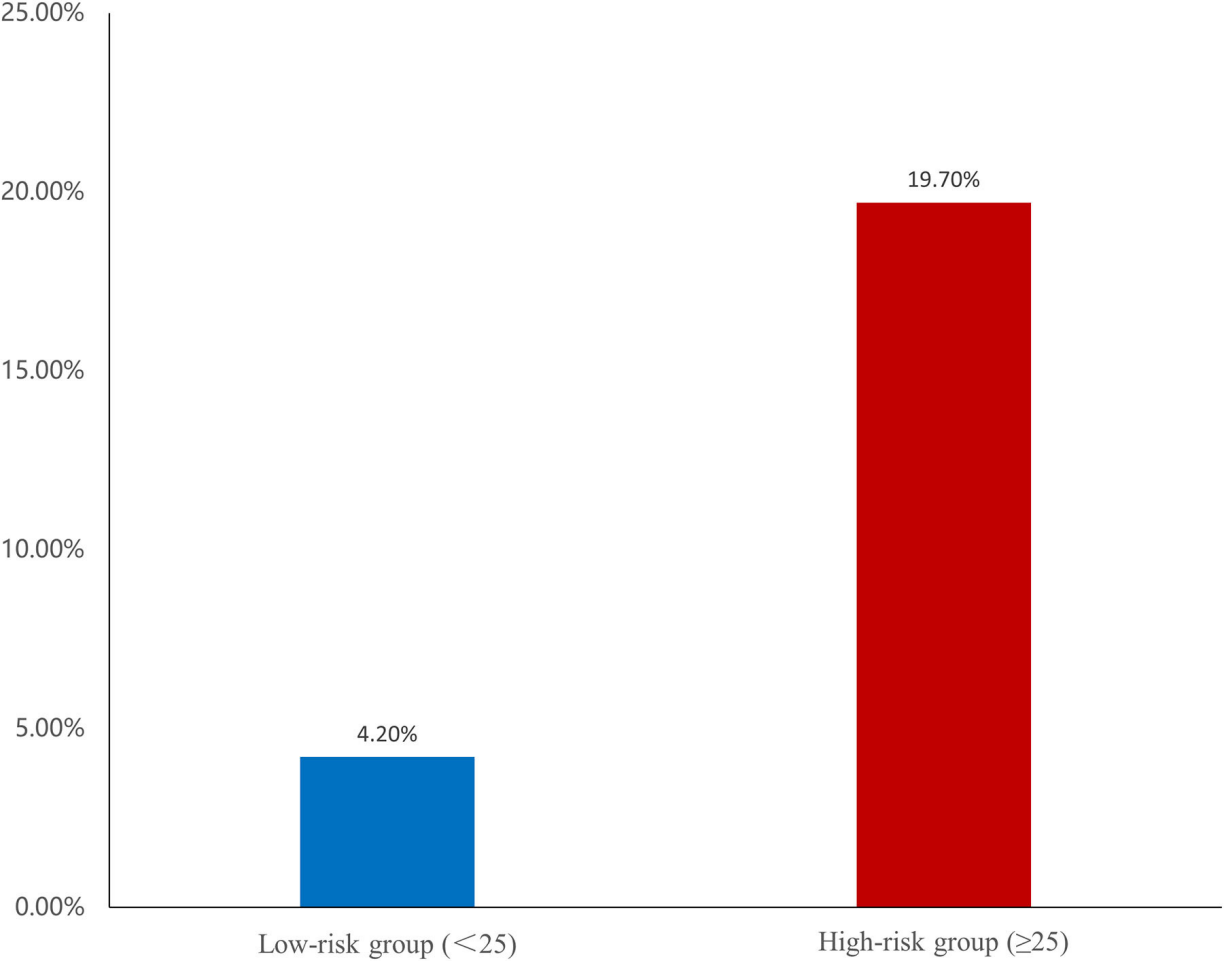
Risk of ventricular tachycardia/ventricular fibrillation (VT/VF) among two groups. The risk classes were categorized based on the cut-off value of FSS_caFFR_.

The area under the curve (AUCs) of SS and FSS_caFFR_ were 0.695 and 0.759, respectively, and both showed statistically significant differences (*p* < 0.001). Additionally, FSS_caFFR_ demonstrated better discriminative power than SS, with a z-statistic of 6.349 (*p* < 0.0001) (Figure 2). Furthermore, we divided the participants into two score categories: the low-risk group (scores <25, risk = 4.20%) and the high-risk group (scores ≥ 25, risk = 19.70%) according to the cutoff value of FSS_caFFR_ (Figure 3). The sensitivity and specificity were 67.2% and 74.2%, respectively (Table 4).

**Table 4:** The best cut-off value, sensitivity, specificity, positive predict value, negative predict value, positive likelihood ratio, negative likelihood ratio of SS and FSS_caFFR_.

	**Cut-off**	**Sensitivity (%)**	**Specificity (%)**	**PPV (%)**	**NPV (%)**	**PLR**	**NLR**
SS	30	58.6	72.6	17.3	94.7	2.139	0.570
FSS_caFFR_	25	67.2	74.2	20.3	95.9	2.605	0.442

*SS, SYNTAX score; FSS_caFFR_, functional SYNTAX score guided by computational pressure-flow dynamics-derived fractional flow reserve; PPV, positive predict value; NPV, negative predict value; PLR, positive likelihood ratio; NLR, negative likelihood ratio*.

### Relationship Between FSS_caFFR_ and Malignant Arrhythmia in Different Periods of AMI

Of the 58 patients with VT/VF, 39 developed VT/VF within 0–48 h after the onset of AMI, while 19 patients were assigned to the late-VT/VF group. Table 5 shows the univariate and multivariate logistic regression analysis results for the correlation factors of both categories of VT/VF. The significant correlation factors of early-VT/VF were STEMI and SBP, whereas those of late-VT/VF were worse Killip class, low LVEF, and long MI-PCI time interval. Finally, FSS_caFFR_ and WBC were significant correlation factors in both the early- and late-VT/VF groups.

**Table 5:** Characteristics of patients with early-VT/VF or late-VT/VF: univariate and multivariate analyses of the predictors in both groups.

	**Non-VT/VF (*n* = 647)**	**Early-VT/VF (*n* = 39)**	**Univariate analysis**	**Multivariate analysis**			**Late-VT/VF (*n* = 19)**	**Univariate analysis**	**Multivariate analysis**		
			***p***	**Or**	**95%CI**	**p**		***p***	**Or**	**95%CI**	***p***
Age (years)	58 ± 13	62 ± 14	0.108				62 ± 14	0.213			
Male (%)	546 (82.8)	29 (74.4)	0.181				14 (73.7)	0.305			
SBP (every increase 10 mmHg)	128 ± 20	111 ± 24	<0.001	0.957	0.938–0.977	<0.001	119 ± 18	0.045			
DBP (every increase 10 mmHg)	80 ± 14	70 ± 19	<0.001				73 ± 9	<0.001			
Heartrate (bpm)	80 ± 15	89 ± 21	<0.001				98 ± 26	<0.001			
BMI (kg/m^2^)	24.4 ± 3.0	24.1 ± 2.6	0.521				25.4 ± 2.8	0.160			
Diabetes mellitus (%)	195 (30.1)	14 (35.9)	0.449				13 (68.4)	0.001			
Hypertension (%)	368 (56.9)	19 (48.7)	0.320				9 (47.4)	0.412			
Dyslipidemia (%)	295 (45.6)	20 (51.3)	0.490				9 (47.4)	0.878			
COPD (%)	20 (3.1)	1 (2.6)	0.853				0 (0)	0.998			
PAD (%)	61 (9.4)	7 (17.9)	0.090				1 (5.3)	0.545			
Smoking (%)	380 (58.7)	23 (59.0)	0.976				10 (52.6)	0.596			
Killipclass>I on admission (%)	137 (21.2)	22 (56.4)	<0.001				17 (89.5)	<0.001	8.621	1.613–45.455	0.012
STEMI (%)	391 (60.4)	36 (92.3)	0.001	6.369	1.751–23.255	0.005	13 (68.4)	0.484			
Peak CK-MB (U/L)	27.695 (5.445–121.880)	155.920 (50.690–273.000)	<0.001				33.710 (18.170–131.820)	0.927			
Peak CTnI (ng/ml)	19.378 (3.526–50.000)	50.000 (17.067–50.000)	0.001				37.451 (1.516–50.000)	0.479			
Peak NT-proBNP (every increase 100 pg/ml)	864 (230–2520)	1822 (787–4231)	0.290				81.050 (48.780–195.680)	0.021			
WBC (×10^9^/L)	10.59 ± 3.49	14.36 ± 6.56	<0.001	1.176	1.088–1.271	<0.001	12.92 ± 3.92	0.005	1.347	1.130–1.606	0.001
Hemoglobin (g/L)	141 ± 50	138 ± 20	0.760				132 ± 25	0.175			
LDL-c (mmol/L)	3.32 ± 1.06	3.35 ± 1.05	0.873				3.75 ± 2.05	0.094			
HDL-c (mmol/L)	1.05 ± 0.27	1.04 ± 0.25	0.694				1.08 ± 0.35	0.623			
Cholesterol (mmol/L)	4.97 ± 1.31	4.88 ± 1.26	0.670				5.39 ± 2.36	0.189			
CRP (mg/L)	4.185 (1.605–12.340)	3.580 (1.600–12.210)	0.831				14.160 (8.650–26.340)	0.020			
Uric acid (μmol/L)	381 ± 112	445 ± 153	0.001				466 ± 122	0.001			
HbAlc (%)	6.0 (5.7–6.8)	6.0 (5.8–6.6)	0.942				6.9 (6.0–8.4)	0.512			
K^+^ (every increase 0.1 mmol/l)	4.01 ± 0.47	4.11 ± 0.59	0.199				4.28 ± 0.49	0.013			
Mg^2+^ (every increase 0.1 mmol/l)	0.84 ± 0.10	0.91 ± 0.23	0.001				0.88 ± 0.11	0.077			
SCr (μmol/L)	100 ± 87	103 ± 35	0.829				122 ± 36	0.315			
eGFR (ml/min/1.73 m^2^)	83 ± 27	71 ± 25	0.012				58 ± 21	<0.001			
LVEDD (mm)	45 ± 4	45 ± 5	0.921				49 ± 7	<0.001			
LVEF (%)	57.7 ± 7.8	51.8 ± 8.1	<0.001				41.0 ± 9.7	<0.001	0.862	0.800–0.929	<0.001
LVA (%)	59 (9.1)	5 (12.8)					6 (31.6)	0.003			
MI-PCI TIME			<0.001					0.010	2.248	1.278–3.955	0.005
<3 h	82 (12.7)	11 (28.2)					0 (0)				
3–12 h	246 (38.0)	23 (59.0)					3 (15.8)				
12–24 h	93 (14.4)	2 (5.1)					5 (26.3)				
24–72 h	88 (13.6)	3 (7.7)					4 (21.1)				
>72 h	138 (21.3)	0 (0)					7 (36.8)				
β-blocker (%)	446 (68.9)	22 (56.4)	0.106				8 (42.1)	0.018			
CCB (%)	57 (8.8)	2 (5.1)	0.432				1 (5.3)	0.594			
ACEIs/ARBs (%)	289 (44.7)	12 (30.8)	0.094				3 (15.8)	0.021			
Diuretics (%)	123 (19.0)	17 (43.6)	<0.001				15 (78.9)	<0.001			
Infarct related artery (%)											
LAD	328 (50.7)	22 (56.4)	0.489				10 (52.6)	0.868			
LCX	100 (15.5)	4 (10.3)	0.383				4 (21.1)	0.510			
RCA	201 (31.1)	12 (30.8)	0.969				5 (26.3)	0.659			
Other	22 (3.4)	2 (5.1)	0.571				0 (0)	0.998			
LMCA disease (%)	59 (9.1)	6 (15.4)	0.200				9 (47.4)	<0.001			
PostTIMI grade <3 (%)	26 (4.0)	4 (10.3)	0.075				4 (21.1)	0.002			
Rentrop grade>1 (%)	87 (13.4)	6 (15.4)	0.732				6 (31.6)	0.032			
Multivessel CAD (%)	479 (74.0)	27 (71.1)	0.444				18 (94.7)	0.098			
Multivessel PCI (%)	79 (12.2)	4 (10.3)	0.665				3 (15.8)	0.684			
Stents used per patient	1.09 ± 0.51	1.08 ± 0.42	0.070				1.11 ± 0.66	0.649			
SS	24.4 ± 11.7	29.2 ± 11.1	0.014				40.3 ± 15.3	<0.001			
FSS_caFFR_	18.0 ± 7.6	28.0 ± 11.2	<0.001	1.055	1.024–1.087	<0.001	39.7 ± 15.0	<0.001	1.052	1.007–1.099	0.023

*SBP, systolic blood pressure; DBP, diastolic blood pressure; BMI, body mass index; COPD, chronic obstructive pulmonary disease; PAD, peripheral artery disease; STEMI, ST-segment elevation myocardial infarction; eGFR, estimated glomerular filtration rate; WBC, white blood cell; CK-MB, creatine kinase-myocardial band; cTnI, cardiac troponin I; NT-proBNP, N-terminal precursor brain natriuretic peptide; WBC, white blood cell; LDL-C, low-density lipoprotein cholesterol; HDL-C, high-density lipoprotein cholesterol; CRP, C-reactive protein; HbAlc, glycated hemoglobin; K^+^, potassium; Mg^2+^, magnesium; SCr, serum creatinine; eGFR, estimated glomerular filtration rate; LVEF, left ventricular ejection fraction; LVEDD, left ventricular end-diastolic dimension; LVA, left ventricular aneurysm; CCB, calcium channel blocker; ACEIs/ARBs, angiotensin-converting enzyme inhibitors/angiotensin receptor blockers; IABP, intra-aortic balloon pump; HD/CRRT, hemodialysis/continuous renal replacement therapy; LAD, left anterior descending; LCX, circumflex; RCA, right coronary artery; LMCA, left main coronary artery; TIMI, thrombolysis in myocardial infarction; CAD, coronary artery disease; PCI, percutaneous coronary intervention; SS, SYNTAX score; FSS_caFFR_, functional SYNTAX score guided by computational pressure-flow dynamics–derived fractional flow reserve*.

## Discussion

The present study is the first to show the correlation between FSS based on caFFR and incident VT/VF in patients with AMI. FSS_caFFR_ was an independent relevant factor of incident VT/VF after AMI. The patients with a higher FSS_caFFR_ of more than 25 showed a higher risk of VT/VF. We also found a significant independent correlation between FSS_caFFR_ and early- and late-onset VT/VF. Overall, our study indicated that patients with AMI with higher FSS_caFFR_ should be alerted to the incident VT/VF, and timely intervention measures should be conducted to prevent VT/VF in clinical practice.

In our study, 8.2% of patients with AMI developed life-threatening VT/VF, a value approximately similar to that reported in the previous studies (1, 7). Some clinical indices associated with incident VT/VF have been recognized in patients with AMI. Consistent with the results of previous studies, patients with STEMI (8–10), patients with lower SBP (1, 11, 12), and patients with reduced LVEF (13) showed an increased risk of incident VT/VF. We found that a higher WBC value showed a higher occurrence rate of VT/VF. Early studies have indicated that, in the acute phase of AMI, neutrophils begin to gather, denature, and infiltrate in the infarcted area under the action of cytokines and chemokines while releasing many inflammatory mediators, reactive oxygen species, and leukotrienes. These events cause the disturbance of local microcirculation and the disorder of cardiac electrophysiological activity, leading to the occurrence of VT/VF (14–16). The positive correlation between ventricular arrhythmias and diuretic use in patients with AMI is also described in the present study (17).

To date, no studies have elaborated on the relationship between SS and VT/VF in patients with AMI. Although SS in the present study was correlated with VT/VF, no statistical significance was found after adjusting for various confounding factors. The AUC value of SS is lower than 0.7, which might be explained by previous studies reporting that many significantly anatomic lesions had no significant functional stenosis (18, 19).

In 2010, the Fractional Flow Reserve vs. Angiography for Multivessel Evaluation (FAME) study demonstrated that FFR-guided PCI could improve the long-term prognosis of patients with multivessel CAD (20). Additionally, FSS as a combination concept of FFR and SS was introduced by Nam et al. (3). The FSS is not only concerned with the anatomic severity but also provided information on coronary physiology. According to the literature, FSS is more instructive in assessing coronary artery conditions and has better prognostic prediction power than SS (21, 22). The caFFR is a new FFR evaluation method. Its assessment is primarily based on angiography images and does not require a pressure wire or hyperemia induction. A recent study has shown that caFFR with FFR as the reference standard has good diagnostic accuracy and consistency (4). Therefore, caFFR has more comprehensive applicable indications than FFR, and it has good clinical application prospects.

Our study is the first to demonstrate that FSS based on caFFR is an independent predictor for VT/VF after AMI. According to the current theory, the occurrence of VT/VF after AMI is not only related to sympathetic excitatory factors caused by acute myocardial ischemia but also associated with abnormal myocardial metabolism, abnormal automaticity, and reentrant formation caused by reperfusion injury in the ischemic area (13). Early studies have shown that the degree of coronary atherosclerosis is more severe in patients with complex coronary artery disease, leading to a weakening of the coronary response to vasodilator factors such as nitric oxide, prostacyclin, and adenosine (23). This finding might explain why patients with AMI with higher FSS_caFFR_ are more likely to develop VT/VF. We showed that the average of FSS_caFFR_ was similar to the SS among the patients with VT/VF, but FSS_caFFR_ was significantly lower than SS among the patients without VT/VF. Grouping patients according to their SS/FSS_caFFR_ can explain this phenomenon more clearly. After calculating FSS_caFFR_, patients whose coronary disease severity was overestimated were reassigned to the lower risk group. Although both SS and FSS_caFFR_ had significant differences in the incidence of VT/VF between the low-risk, medium-risk, and high-risk groups, the difference in the incidence of VT/VF was greater in the FSS_caFFR_ group than in the SS group (Supplementary Figure 2), which indicated that FSS_caFFR_ can better distinguish patients with different levels of severity of CAD. Additionally, ROC analysis showed that FSS_caFFR_ has better discriminative power concerning the incident VT/VF than SS. Thus, SS combining anatomic and functional information is more relevant to incident VT/VF in patients with AMI than anatomic assessments alone.

Early studies have found that the mechanism and clinical characteristics of lethal VT/VF vary at different stages after AMI. The occurrence of VT/VF within 48 h after the onset of AMI may be more related to cardiac electrical and hemodynamic instability (24), while the occurrence of late VT/VF may be more related to overactivated inflammation and cardiac scar formation (25, 26). This finding indicates that the long-term prognosis of patients with early VT/VF may be different from that of patients with late VT/VF. To further investigate the relationship between FSS_caFFR_ and the two types of VT/VF, the patients with VT/VF were categorized into two groups. Our results revealed that high FSS_caFFR_ is an independent risk factor for both early and late VT/VF. Thus, the more severe the functional ischemia of the coronary artery, the more likely it is to cause electrical disturbances, thereby inducing early VT/VF. Similarly, the degree of functional ischemia of the coronary artery will also affect inflammation and the formation of cardiac scars, increasing the incidence of late VT/VF. Therefore, high FSS_caFFR_ indicates that the incidence of VT/VF will increase in both early and late stages after myocardial infarction and extending monitoring time and early intervention may benefit patients with high FSS_caFFR_.

## Limitations

Our study had several limitations. First, this study is a single-center retrospective study, and the study population was relatively small. Therefore, some bias may exist in the study population. Second, because of the limitation of retrospective data, our study did not consider the factor of microcirculation dysfunction during the period of AMI that might temporarily increase FFR of the coronary artery slightly (27, 28), thereby affecting the calculation of FSS_caFFR_. However, notably, the current mainstream condition to determine coronary ischemia in patients with AMI is still FFR ≤0.8 (29). Therefore, we believe that the results of this study are still reasonable and reliable. Third, the aortic root pressure was not measured in real-time, and it was obtained from the interventional database. Additionally, the hemodynamics of patients with AMI were very unstable. Therefore, it might slightly influence the results of caFFR because a real-time measured aortic root blood pressure can help obtain a more accurate result of caFFR (30). We believe that more prospective studies are needed to verify these findings in the future.

## Conclusion

The present study is the first to comprehensively examine the relationship between the VT/VF and the severity of CAD using SS and FSS_caFFR_ in patients with AMI. Our results showed that FSS_caFFR_ has a higher correlation with VT/VF than SS. Additionally, FSS_caFFR_ was an independent correlation factor of VT/VF, regardless of whether VT/VF occurred in the early or late stage after AMI.

## Data Availability Statement

The original contributions presented in the study are included in the article/Supplementary Material, further inquiries can be directed to the corresponding author/s.

## Ethics Statement

The studies involving human participants were reviewed and approved by the Ethics Committee of the Southern Medical University. Written informed consent for participation was not required for this study in accordance with the national legislation and the institutional requirements.

## Author Contributions

JP and JX contributed to the conception or design of the work. JP, QZ, LL, YC, GL, HL, JL, XZ, YT, JP, YY, and DM contributed to the acquisition, analysis, or interpretation of data for the work. JP drafted the manuscript. JP, QZ, LL, and JX critically revised the manuscript. All authors take responsibility for all aspects of the reliability and freedom from bias of the data presented and their discussed interpretation, gave final approval and agreed to be accountable for all aspects of work, ensuring integrity, and accuracy.

## Funding

This work was supported by the National Key R&D Program of China (No. 2018YFC1312803), National Natural Science Foundation (No. 81974266), and the Key Program of Zhengcheng Branch of Nanfang Hospital.

## Conflict of Interest

The authors declare that the research was conducted in the absence of any commercial or financial relationships that could be construed as a potential conflict of interest.

## Publisher's Note

All claims expressed in this article are solely those of the authors and do not necessarily represent those of their affiliated organizations, or those of the publisher, the editors and the reviewers. Any product that may be evaluated in this article, or claim that may be made by its manufacturer, is not guaranteed or endorsed by the publisher.

## Supplementary Material

Supplementary Figure 1 Proportions of the study population according to the tertiles of the SS.

Supplementary Figure 2 Incidence of VT/VF between low-risk, medium-risk, and high-risk groups of SS and FSS_caFFR_.

Supplementary Table 1 Angiographic date of patients in the non-VT/VT group and the VT/VF group.

## Abbreviations

AMI: acute myocardial infarction
ACEIs/ARBs: angiotensin-converting enzyme inhibitors/angiotensin receptor blockers
BMI: body mass index
CABG: coronary artery bypass surgery
CAD: coronary artery disease
caFFR: coronary angiography-derived FFR
CCB: calcium channel blocker
CK-MB: myocardial creatine kinase
COPD: chronic obstructive pulmonary diseases
CRP: C-reactive protein
CTnI: cardica troponin I
DBP: diastolic blood pressure
eGFR: estimated glomerular filtration rate
FFR: fractional flow reserve
FSS: functional SYNTAX score
FSScaFFR: functional SYNTAX score guided by computational pressure-flow dynamics derived fractional flow reserve
HD/CRRT: hemodialysis/continuous renal replacement therapy
HDL-c: High-density lipoprotein cholesterol
IABP: intra-aortic balloon pump
LDL-c: low-density lipoprotein cholesterol
LMCA: left main stem disease
LVA: left ventricular aneurysm
LVEDD: left ventricular end-diastolic dimension
LVEF: left ventricular ejection fraction
NT-proBNP: N-terminal precursor brain natriuretic peptide
PAD: peripheral arterial disease
PCI: percutaneous coronary intervention
ROC: receiver operator characteristic curves
SBP: systolic blood pressure
SCr: creatinine
STEMI: ST-segment elevation myocardial infarction
SS: SYNTAX score
SYNTAX: synergy between percutaneous coronary intervention with taxus and cardiac surgery
TIMI: thrombolysis in myocardial infarction
VT/VF: ventricular tachycardia/ventricular fibrillation
WBC: white blood cell.
