# Supplementary material for: Impact of the caFFR-Guided Functional SYNTAX Score on Ventricular Tachycardia/Fibrillation Development in Patients With Acute Myocardial Infarction
Source: Front Cardiovasc Med. 2022 Apr 12;9:807805. doi: 10.3389/fcvm.2022.807805 (PMC9040892; doi:10.3389/fcvm.2022.807805)
Supplement: Supplementary Table 1 — Angiographic date of patients in the non-VT/VT group and the VT/VF group. [file Table_1.DOCX]

**Table S1** Angiographic date of patients in non-VT/VT group and VT/VF group.

|  | non-VT/VF group | VT/VF group | p |
| --- | --- | --- | --- |
| Number of lesions per patient | 2.88±0.06 | 3.21±0.2 | 0.043 |
| Lesion location |  |  | 0.008 |
| -LMCA | 59(3.2) | 15(8.1) |  |
| -LAD | 657(35.3) | 64(34.4) |  |
| -LCX | 484(26.0) | 44(23.7) |  |
| -RCA | 662(35.5) | 63(33.8) |  |
| Features of Lesions |  |  | 0.189 |
| -50-70% narrowing | 272(14.6) | 22(11.8) |  |
| -70-90% narrowing | 561(30.2) | 50(26.9) |  |
| -90-99% narrowing | 621(33.3) | 61(32.8) |  |
| -Total occlusion | 408(21.9) | 53(28.5) |  |
| Bifurcation lesion | 527(28.3) | 57(30.6) | 0.500 |
| Severe tortuosity | 69(3.7) | 2(1.1) | 0.062 |
| Lesion length > 20mm | 480(25.8) | 55(29.6) | 0.262 |
| Severe calcification | 19(1.0) | 1(0.5) | 1.000 |
| Aorto Ostial lesion | 117(6.3) | 16(8.6) | 0.221 |

LMCA: left main coronary artery; LAD: left anterior descending; LCX: circumflex; RCA: right coronary artery;
